# Dehydroepiandrosterone inhibits the progression phase of mammary carcinogenesis by inducing cellular senescence via a p16-dependent but p53-independent mechanism

**DOI:** 10.1186/bcr1350

**Published:** 2005-11-16

**Authors:** Anne Shilkaitis, Albert Green, Vasu Punj, Vernon Steele, Ronald Lubet, Konstantin Christov

**Affiliations:** 1 Department of Surgical Oncology, University of Illinois, 840 S. Wood Str., Chicago, IL 60612, USA; 2 National Cancer Institute, Division of Cancer Prevention, 6130 Executive Blvd., Bethesda, MD 20892, USA

## Abstract

**Introduction:**

Dehydroepiandrosterone (DHEA), an adrenal 17-ketosteroid, is a precursor of testosterone and 17β-estradiol. Studies have shown that DHEA inhibits carcinogenesis in mammary gland and prostate as well as other organs, a process that is not hormone dependent. Little is known about the molecular mechanisms of DHEA-mediated inhibition of the neoplastic process. Here we examine whether DHEA and its analog DHEA 8354 can suppress the progression of hyperplastic and premalignant (carcinoma *in situ*) lesions in mammary gland toward malignant tumors and the cellular mechanisms involved.

**Methods:**

Rats were treated with *N*-nitroso-*N*-methylurea and allowed to develop mammary hyperplastic and premalignant lesions with a maximum frequency 6 weeks after carcinogen administration. The animals were then given DHEA or DHEA 8354 in the diet at 125 or 1,000 mg/kg diet for 6 weeks. The effect of these agents on induction of apoptosis, senescence, cell proliferation, tumor burden and various effectors of cellular signaling were determined.

**Results:**

Both agents induced a dose-dependent decrease in tumor multiplicity and in tumor burden. In addition they induced a senescent phenotype in tumor cells, inhibited cell proliferation and increased the number of apoptotic cells. The DHEA-induced cellular effects were associated with increased expression of p16 and p21, but not p53 expression, implicating a p53-independent mechanism in their action.

**Conclusion:**

We provide evidence that DHEA and DHEA 8354 can suppress mammary carcinogenesis by altering various cellular functions, inducing cellular senescence, in tumor cells with the potential involvement of p16 and p21 in mediating these effects.

## Introduction

Dehydroepiandrosterone (DHEA) is a 17-ketosteroid that is produced at high levels in the adrenals of primates and is the chief precursor of androstenedione, which itself is readily converted into testosterone and 17β-estradiol at the tissue level [1]. Epidemiological data have shown that premenopausal women with high levels of DHEA in the serum develop less breast carcinomas than those with low levels of DHEA [2]. In postmenopausal women DHEA has been hypothesized to contribute to the increased incidence of breast cancer [3]. Studies on animal model systems revealed that DHEA is a powerful inhibitor of mammary, prostate, skin, lung, liver, and thyroid carcinogenesis [4-7]. DHEA and its fluorinated analogue, DHEA 8354, given after carcinogen administration, inhibited mammary carcinogenesis in rats in a dose-dependent manner [6,7]. DHEA combined with 4-(Hydroxyphenyl)retinamide (4-HPR) further decreased the incidence and frequency of mammary tumors in an *N*-nitroso-*N*-methylurea (MNU) carcinogenesis model [4]. Tamoxifen (0.08 mg/kg diet) combined with low-dose DHEA (400 mg/kg diet) further decreased the incidence and multiplicity of mammary tumors [6]. DHEA also decreased the incidence of mammary tumors in rats injected with dimethylbenz(a)anthracene (DMBA) into mammary gland parenchyma and continuously stimulated with prolactin [8]. Studies with C3(1)/SV40 Tag mice, which can spontaneously develop estrogen receptor (ER)-negative mammary and prostate tumors, have shown that DHEA at 4,000 mg/kg diet given between the 7 th and 19 th weeks of age, decreased the incidence of mammary tumors by 30% and tumor multiplicity by about 50% [9]. Furthermore, in both DHEA-treated C3(1)/SV40 Tag mice and DHEA-treated rats the circulating estradiol levels were increased, suggesting the involvement of a tumor suppressor mechanism that is not directly related to the ER signaling. DHEA was also effective in inhibiting the growth of ZR-75-1 ER-positive breast cancer cells transplanted into nude mice [10]. In most of these studies, DHEA was given in the diet 1 week before and/or continuously after carcinogen administration, until the animals were killed [4-6]. However, with this approach the limited number of tumors that occur in DHEA-treated animals are apparently resistant to DHEA and therefore the biomarkers that would be identified might not reflect the preventive and antitumor potential of DHEA in the course of tumor development. Further, the molecular mechanism of action of DHEA remains poorly defined.

Here we have examined the effect of DHEA and its analog DHEA 8354, initiated 6 weeks after carcinogen administration, when hyperplastic and premalignant lesions occur in the mammary gland, on the following: first, the incidence, multiplicity and weight (burden) of mammary tumors; second, the proliferative activity of tumor cells; third, apoptosis; fourth, cellular senescence; fifth, cell cycle distribution; and sixth, the expression of p53, p21, and p16. To identify senescent cells in mammary tissues and tumors, a panel of methods was employed, including senescence-associated β-galactosidase (SA-β-Gal) staining [11], continuous labeling with bromodeoxyuridine (BrdU) [12], analysis of 90° light scatter by flow cytometry, and cytomorphological criteria [13]. Because p53, p21, and/or p16 genes are key mediators for the initiation and maintenance of cellular senescence and apoptosis [14-17], their role in such mechanisms was investigated. It has been recently observed that p16^INK4A ^is overexpressed in senescent cells, suggesting that it might be upregulated in mammary tumors treated with DHEA [18]. We provide comprehensive evidence of a correlation between the inhibitory effects of DHEA on mammary carcinogenesis, induction of cellular senescence, inhibition of cell proliferation, induction of apoptosis and the expression of p21 and p16^INK4A^.

## Materials and methods

### Animals

Female, virgin Sprague-Dawley (Hsp: (SD/BR)) rats were obtained from Harlan Sprague-Dawley (Indianapolis, IN, USA) at 35 days of age; after 1 week of quarantine they were randomized by weight and injected with carcinogen. Beginning 3 weeks after carcinogen administration, the animals were palpated weekly to monitor tumor appearance in the mammary gland. The animals were fed with 4% Purina diet chow *ad libitum *and had free access to water. Rats were weighed weekly and checked daily for any sign of toxicity.

### Chemical carcinogen

MNU was obtained from Ash Stevens Inc. (Detroit, MI, USA), dissolved in sterile acidified saline (pH 5.0), and injected intraperitoneally (50 mg/kg body weight) when the animals were 50 days old. Control animals at the same age received sterile saline only.

### DHEA and DHEA 8354

DHEA was obtained from Sigma Chemical Co. (St Louis, MO, USA). AHEA 8354 was supplied by the NCI repository (Bethesda, MD, USA). Both agents were mixed in AIN-76A purified mice/rat diet (Teklad, Madison, WI, USA) to achieve a concentration of 125 or 1,000 mg/kg diet. Both agents were given for 6 weeks, starting 6 weeks after the administration of carcinogen [19].

### Mammary tumors

The growth of mammary tumors was monitored by measuring their length (*L*), width (*W*), and thickness (*T*). Tumor volume (*V*_T_) was calculated from the formula *V*_T _= *LWT*/2. Animals were killed by CO_2 _asphyxiation; the tumors were excised, weighed, and cut into halves. One half was fixed in 10% neutral formalin for histomorphology, BrdU, and apoptosis assay; the other half was frozen in liquid nitrogen and stored at -80°C for the assessment of senescence-like cells by SA-β-Gal staining, for the assessment of cell cycle distribution by flow cytometry, and for western blotting.

### Cell proliferation

Two sets of experiments were performed. In the first, animals were injected intraperitoneally with BrdU (50 mg/kg body weight; Sigma) 2 hours before being killed and the percentage of BrdU-labeled tumor cells was examined. In the second experiment, animals with palpable mammary tumors were continuously labeled with BrdU (7 days) by means of osmotic pumps (Anza Co., Palo Alto, CA, USA) implanted subcutaneously. The BrdU-labeled cells in both studies were detected by an anti-BrdU monoclonal antibody (Becton Dickinson, Palo Alto, CA, USA) and an ABC kit (Vector, Burlingame, CA, USA), as described previously [20].

### Apoptosis

Apoptotic cells were identified on parallel tissue sections by TUNEL (TdT-mediated dUTP nick end labelling) assay, as recommended in the ApopTag *in situ *hybridization detection kit (Oncor Co., Gaithersburg, MD, USA).

### Cell cycle arrest

Frozen tumor samples (100 to 200 mg) were disintegrated mechanically and enzymatically to generate a cell suspension as described previously [21]. The cells were stained by propidium iodide (50 μg/ml) and the DNA amount in the cells and the cell cycle distribution were analyzed with a FACS Coulter counter (EPICS Elite 5, Coulter Co., Miami, FL, USA).

### Identification of senescent cells in mammary tumors

The SA-β-Gal activity assay was employed for the identification of senescent cells in mammary tissues and tumors [11]. Frozen sections (5 to 7 μm thick) from control animals and sections of tumors from animals treated with DHEA and DHEA 8354 were fixed in 3.0% formaldehyde for 5 minutes, washed in phosphate-buffered saline and stained in X-Gal (5-bromo-4-chloro-3-indolyl-β-D-galactoside; Sigma) solution at pH 6.0 for 24 hours at 37°C, following our protocol described previously [12].

### Expression of p53, p21, and p16

The expression of these genes was assessed by western blotting and immunocytochemical staining (ICH). For western blotting, frozen tumor tissue (about 100 mg) was disintegrated with a Sonifer Cell Disruptor in lysis buffer containing a cocktail of protease inhibitors (Roche Biochemicals); 50 to 100 μg of the isolated protein was separated by electrophoresis in a 10 to 20% Tris-glycine gel and transblotted to Immobilon-P membranes (Millipore, Bedford, MA, USA). Blots were probed with primary antibody and corresponding secondary antibody. Antibodies against p53 and p16 were purchased from Santa Cruz Biotech Inc. (Santa Cruz, CA, USA), and antibodies against p21 were obtained from R&D Systems Inc., Minneapolis, MN.

For ICH, formalin-fixed paraffin-embedded tissue samples were used. The same antibodies that were employed for western blotting were also used for ICH. ABC kit and 3,3'-Diaminobenzidine (Sigma) staining were used to identify cells expressing the corresponding antigens. The percentage of cells positive for p16 or p21 was calculated after counting more than 1,000 cells. For quantitative analysis of cells stained with p21 and p16, we first assessed the percentage of cell nuclei positively stained for the above antigens and scored the samples from 0 to 3 as follows: 0, 0 to 5%; 1, 6 to 20%, 2, 21 to 50%; 3, more than 50%. Then we assessed the intensity of staining by using the same 0 to 3 grading system: 0, none (similar to background staining); 1, weak; 2, moderate; 3, strong. The data from each score from each tumor were multiplied and the result was used to assess the values for each group. Two pathologists (AS and KC) independently evaluated the samples and the difference in scores was in the range 0 to 20%. p16 and p21 values were generated by employing a semi-quantitative approach (0 to 3) for positively stained cells multiplied by the intensity of staining (0 to 3).

### Statistical analysis

Comparisons of tumor incidence curves for treated and control animals were made with a life table analysis and the log-rank test. Tumor multiplicity data were compared by using Armitage's test for trend in proportion. Body and tumor weight data were compared with the two-tailed Fisher *t *test by using analysis of variance [22].

## Results

### DHEA and DHEA 8654 suppress the progression phase of mammary carcinogenesis

**Figure 1:**
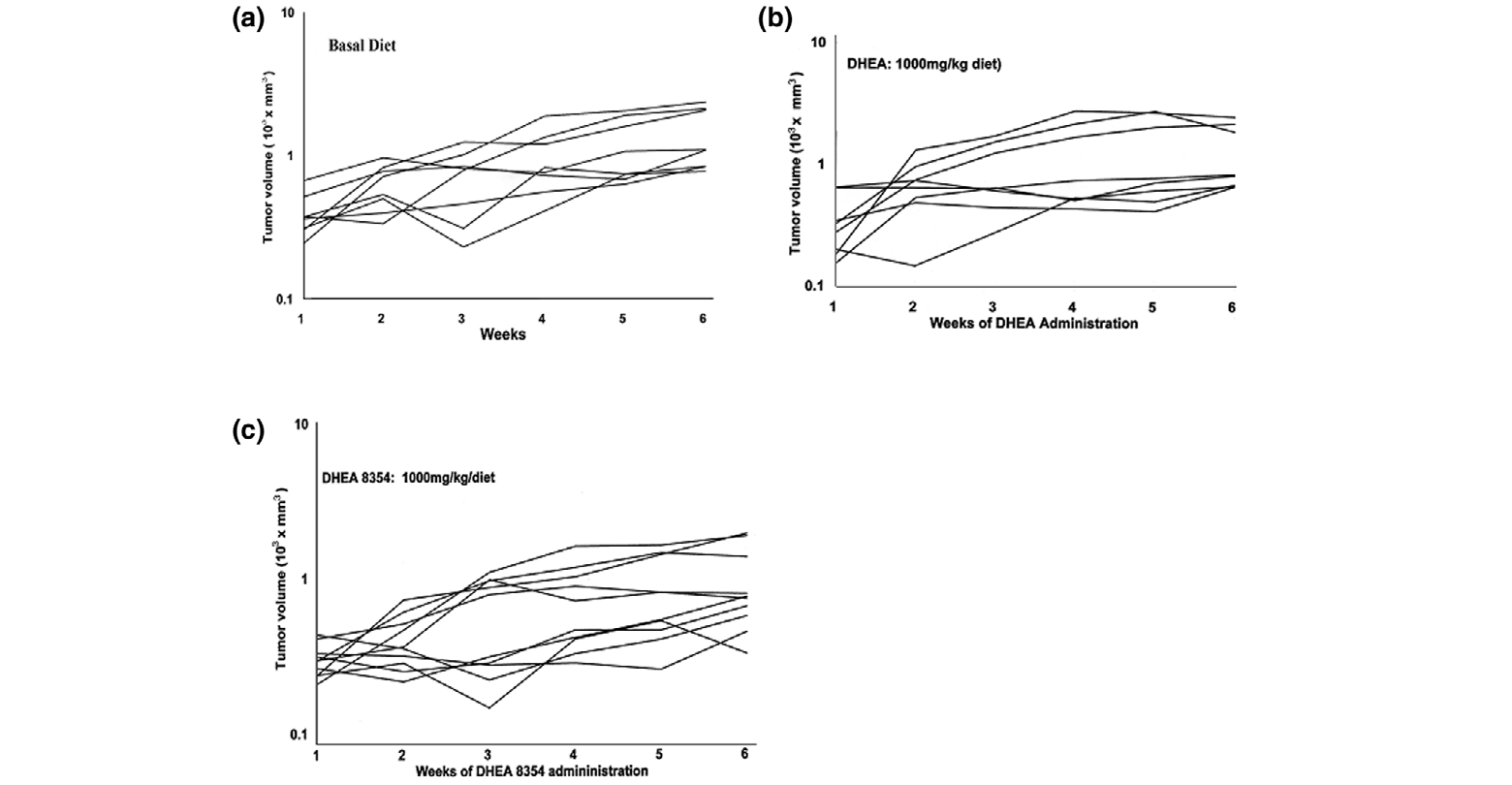
DHEA and DHEA8354 suppress the growth of mammary tumors. **(a) **Growth curves of mammary tumors in control animals. **(b, c) **Growth curves of mammary tumors in animals treated with dehydroepiandrosterone (DHEA) (b) or DHEA 8354 (c) (1,000 mg/kg in diet). In 50% of DHEA-treated tumors a net decrease in growth potential over control was observed, whereas with DHEA 8354 some animals showed a decrease in growth potential. Each curve represents an individual tumor.

To examine the effects of DHEA and DHEA 8354 on the progression of these lesions towards malignant tumors, both agents were added to the diet starting 6 weeks after the administration of carcinogen. The high doses of DHEA and DHEA 8354 only decreased the growth potential in some mammary tumors (Fig. 1). DHEA and DHEA 8354 given for 6 weeks at low (125 mg/kg diet) or high (1,000 mg/kg diet) dose did not affect the animals' body weight or the incidence of tumors but decreased tumor multiplicity (Table 1): from 4 tumors per animal in the control group to 3.2 and 2.3 in the low-dose and high-dose DHEA groups (*p *< 0.05), respectively. DHEA 8354 was less effective: the decrease in tumor multiplicity was only 15% at the high dose. The high doses of both agents also suppressed the tumor burden, from 5.7 g per animal in control animals to 2.3 g (*p *< 0.01) and 2.6 g (*p *< 0.01) per animal in animals treated with DHEA and DHEA 8354, respectively. Morphological examination of tumors revealed that the high doses of DHEA or DHEA 8354 induced partial disintegration of tumor parenchyma. The low doses (125 mg/kg diet) were less efficacious and did not affect tumor morphology.

### DHEA and DHEA 8354 decreased cell proliferation and induced apoptosis in mammary tumors

**Figure 2:**
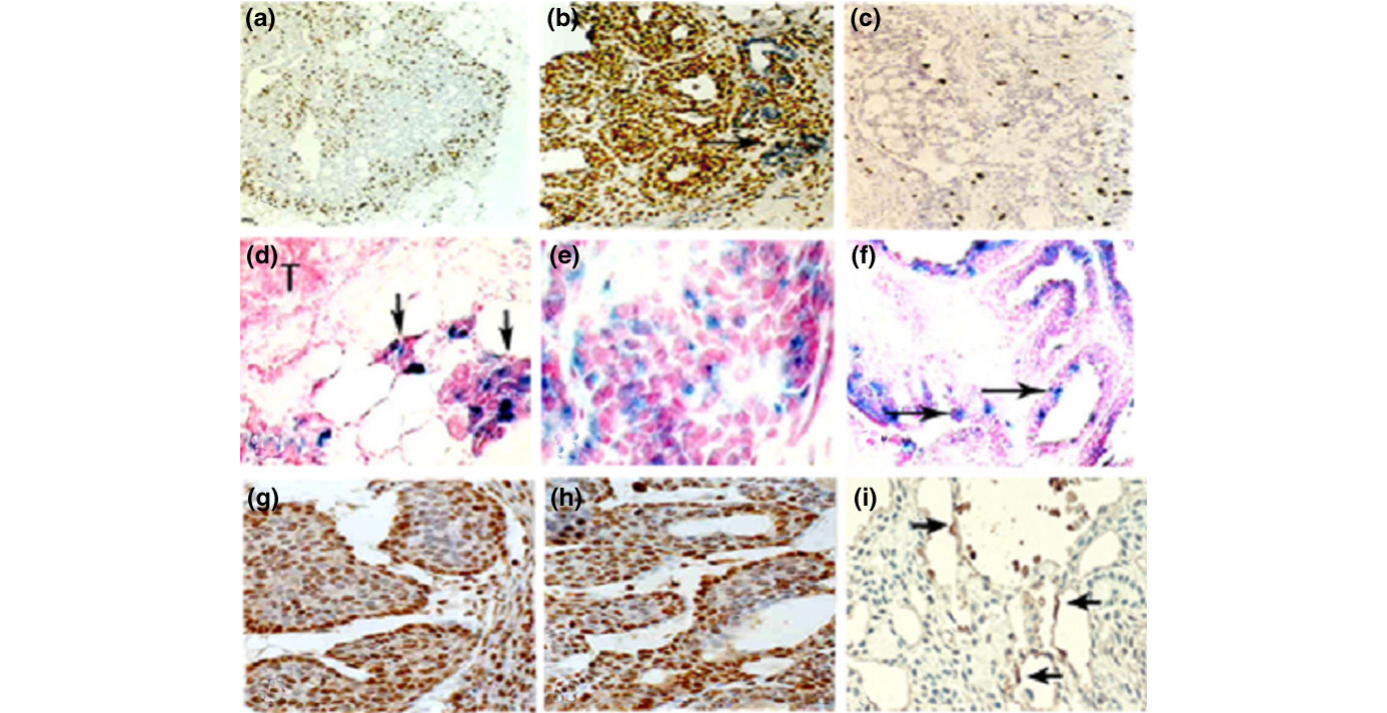
DHEA and DHEA8354 induce cellular senescence, inhibit cell proliferation and increase apoptosis in mammary tumors. **(a-c) **Cell proliferation in control mammary tumors (a,b) and in tumors treated with dehydroepiandrosterone (DHEA) (c). DHEA significantly decreased the proportion of bromodeoxyuridine-labeled cells (c). The slide was counterstained with hematoxylin; original magnification × 100. **(d-f) **DHEA-induced cellular senescence (SA-β-Gal-stained cells) in normal lobular cells (d, arrows) and in mammary tumors (e and f, arrows). **(g,h) **p21 and p16 were expressed in most mammary tumor cells. DHEA also induced tumor disintegration and in these areas p16 (arrows) was preferentially detected in tumor areas where SA-β-Gal-stained cells were also identified.

The percentage of BrdU-labeled cells was variable between individual tumors. Some had more than 20% BrdU-labeled cells (Fig. 2a), whereas others had less than 5% labeled cells. However, the high dose of both agents significantly decreased BrdU-LI, from 15.6 ± 3.8% (SD) in control animals to 2.2 ± 1.2% in DHEA-treated animals (*p *< 0.01) and to 5.3 ± 1.8% in DHEA 8354-treated animals (*p *< 0.01), respectively (Table 2 and Fig. 2c). The low doses of DHEA and DHEA 8354 were less efficacious. DHEA and DHEA 8354 also increased apoptotic cells in mammary tumors. In control tumors apoptotic cells are rarely observed. However, the percentage of apoptotic cells increased from 0.7 ± 0.4% in control animals to 3.2 ± 1.0% in DHEA-treated animals and to 1.5 ± 0.5% in DHEA 8354-treated animals. Low doses of either agent were less efficacious and did not significantly increase apoptotic cells (Table 2).

### DHEA and DHEA 8354 induced cellular senescence in mammary tumors

The cells that developed senescent phenotype were identified by SA-β-Gal staining. In control animals, SA-β-Gal-stained cells were few in mammary tumors (0.4 ± 0.2%) and were localized predominantly in the non-proliferating tumor areas, whereas in the animals treated with DHEA SA-β-Gal cells increased preferentially in normal lobular structures (Fig. 2d) and in tumors. Overall, there were tumors with very few SA-β-Gal-stained cells (Fig. 2d) and tumors with a relatively large number of senescent cells (Fig. 2e,f). Statistically, SA-β-Gal cells increased from 0.4 ± 0.2% in control tumors to 1.9 ± 1.8% (*p *< 0.01) and to 3.3 ± 2.0% (*p *< 0.001) in the animals treated with low and high doses of DHEA, respectively (Table 2). As shown in Fig. 2b, after 7 days of continual BrdU infusion almost all tumor cells were labeled with BrdU except for a few cells, which could be considered to be in replicative senescence (Fig. 2b).

### DHEA and DHEA 8354 arrest tumor cells in the G1/G0 phase of the cell cycle

**Figure 3:**
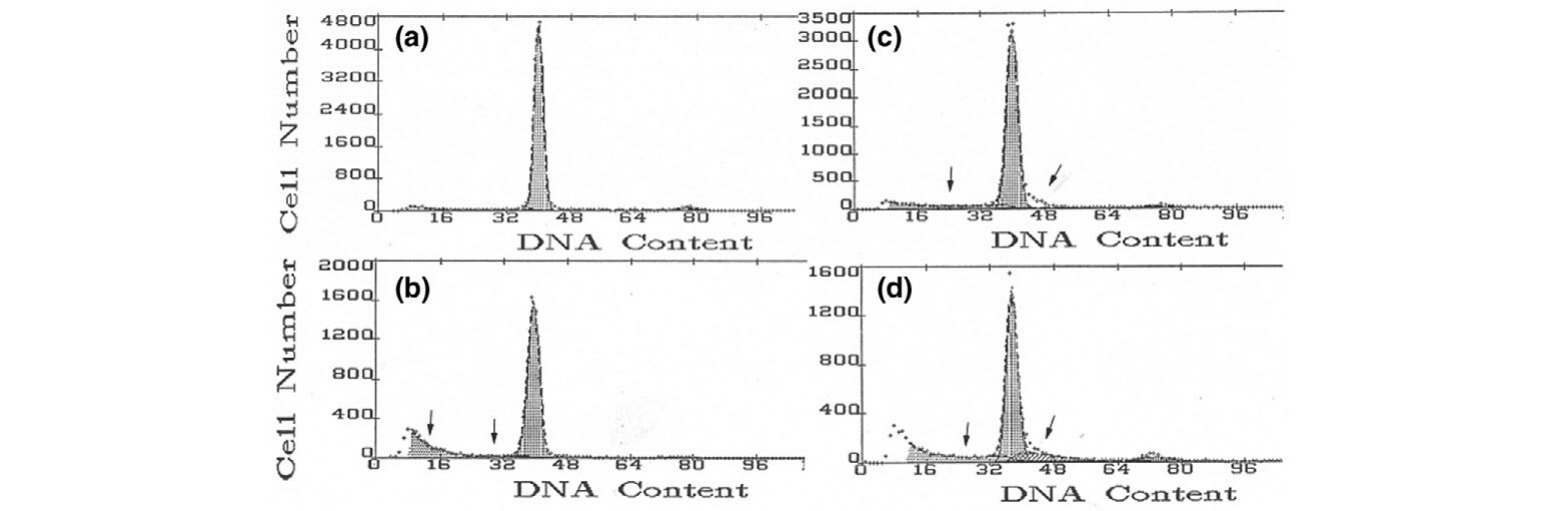
Effects of DHEA and DHEA8354 on the cell cycle. **(a-c) **Cell cycle distribution in MNU-induced mammary tumor of a control animal (a) and in an animal treated with dehydroepiandrosterone (DHEA) (b,c). Control tumors showed a low level of S-phase cells and a lack of cells with hypodiploid values of DNA. DHEA treatment induced an increase in apoptotic cells (b, arrow) and a decrease in G2M phase. A cell population is identified with DNA values after the G1/G0 peak (c, arrow), suggesting DNA abnormalities typical for senescent cells from another mammary tumor from an animal treated with DHEA. **(d) **DHEA induced both apoptosis and senescence (a small area after the G1/G0 peak (arrow), indicative of senescence.

In control tumors (*n *= 10), the percentages of cells in the G0/G1 and S phases were 92.3 ± 0.8% and 5.4 ± 2.2%, respectively, whereas in animals treated with DHEA or DHEA 8354 the S phase decreased to 1.8 ± 0.8% (*p *< 0.001) and 2.1 ± 1.0% (*p *< 0.001), respectively(Table 3). These results are in accord with our cell proliferation data. The decrease in S phase concomitant with an increase in G1/G0 phase suggests that DHEA and DHEA 8354 arrested the cells in the G1/G0 phase of the cycle (Fig. 3a). Individual histograms also showed cells with DNA values below those of the G1/G0 peak (Fig. 3b-d), which represent the apoptotic cells. A small subset of cells were identified in the hyperdiploid and hypertetraploid region of the histograms, which probably represent some of the senescent cells (Fig. 3c,d).

### Mechanism of cellular senescence induced by DHEA and DHEA 8354

**Figure 4:**
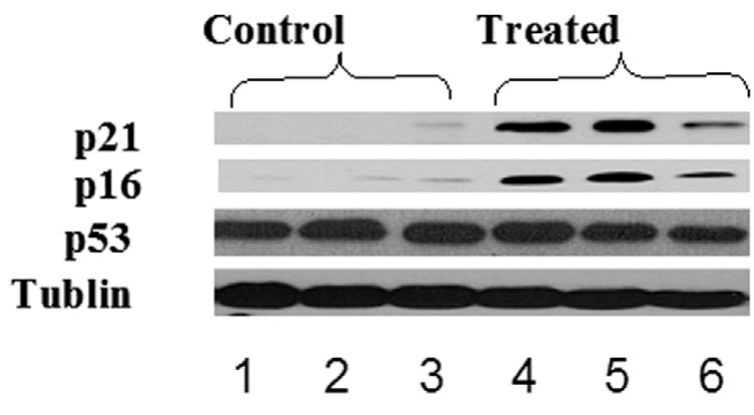
DHEA and DHEA8354 differentially affect p16, p21, and p53 protein expression: Western blot data of control tumors (lanes 1 to 3) and tumors treated with DHEA (1,000 mg/kg diet) (lanes 4 to 6) as estimated by western blotting. For western blotting, tumors were selected with relatively high values of senescence-associated β-galactosidase (SA-β-Gal)-labelled cells and low values of bromodeoxyuridine-labeled cells.

The expression of p53, p21, and p16 was examined by ICH (Fig. 3 and Table 4) and western blotting (Fig. 4). The differences in the levels of p21-stained cells in control animals over those treated with DHEA were not significant (*p *< 0.2), whereas those for p16 were highly significant (*p *< 0.01). The level of p53 remained unaltered in control and DHEA-treated animals; they are therefore not included in Table 4. Figure 2h shows a mammary tumor from a control animal with most cells positively stained for p21; in contrast, p16-stained cells of a mammary tumor from a control animal are shown in Fig. 2i. However, in the animals treated with the DHEA diet we could identify cells positively stained for p16 (Fig. 2i) in the same morphological structures in which SA-β-Gal-stained cells were also identified (compare Fig. 2f with Fig. 2i).

**Figure 5:**
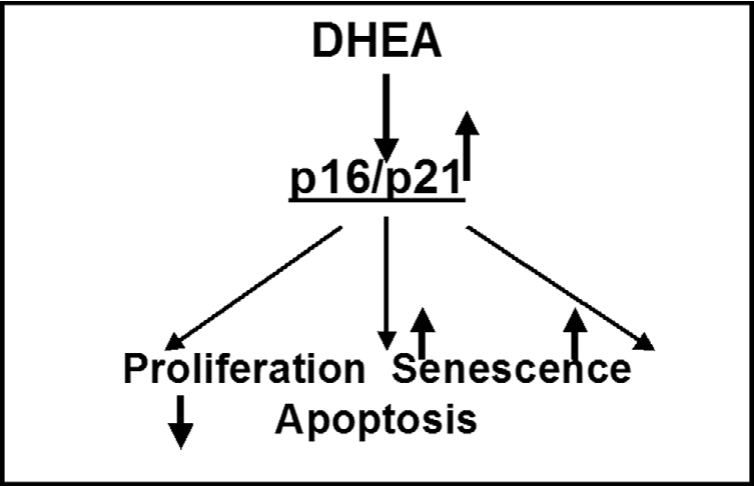
A hypothetical model of inhibition of mammary carcinogenesis by DHEA. Our data indicate that DHEA, by increasing p16 and p21 expression, may suppress cell proliferation and induce senescence and apoptosis. The arrows indicate the changes in the corresponding parameters.

For western blotting, tumors were selected that had high proportions (more than 3.0%) of SA-β-Gal-stained cells. It seems that DHEA given for 6 weeks induced the expression of p16 and p21, but not that of p53, suggesting a p53-independent mechanism of induction of cellular senescence and of cell growth inhibition (Fig. 5).

## Discussion

In a previous study we observed that 5 to 6 weeks after the administration of MNU (by the injection of 50 mg/kg body weight) all Sprague-Dawley rats developed multiple hyperplastic and premalignant lesions in the mammary gland [23]. The main objective of this study was therefore to examine whether DHEA and its analog DHEA 8354 could suppress the progression of hyperplastic and premalignant lesions in the mammary gland towards malignant tumors and to identify the cellular mechanisms involved in this process. The progression phase was considered to be the time between the occurrence of hyperplastic and premalignant (carcinoma *in situ*) lesions in the mammary gland and the development of palpable tumors. We therefore administered DHEA and DHEA 8354 in the diet, starting 6 weeks after MNU administration, when most hyperplastic and premalignant (carcinoma *in situ*) mammary lesions occurred [23]. Both agents were used at doses reported to show an inhibitory effect on mammary carcinogenesis [6,7].

The decrease in tumor multiplicity brought about by DHEA and DHEA 8354 is apparently a consequence of inhibition in the progression of existing hyperplastic and premalignant lesions as well as a decrease in the occurrence of new lesions. The decrease in tumor burden is a complex phenomenon involving the inhibition of cell proliferation and the induction of apoptosis and of cellular senescence. We previously examined several preventive agents for their effects on cell proliferation and apoptosis [19,20]. More recently we included markers of cellular senescence [12], which have been widely used in characterizing the senescent phenotype [24]. To ensure that in control and DHEA-treated mammary tumors there were non-proliferating cells, animals were implanted subcutaneously with osmotic pumps that continuously released BrdU and thus specifically labeled proliferating cells during the S phase of the cell cycle. Because the cell cycle time of mammary tumors in rats is about 18 hours [26] (Christov K, Lubet R, Steele V., Grubbs C., unpublished data), a 7-day delivery of BrdU by means of osmotic pumps would label all cells passing through at least eight or nine consecutive cell cycles, whereas cells in terminal proliferative arrest would remain unlabeled and could be considered senescent [13].

The mechanisms involved in the initiation and maintenance of cellular senescence in tumor cell systems are poorly understood. Most studies on cell lines indicate that the induction and overexpression of wild-type p53 (mostly by cytotoxic agents or irradiation) can cause cell growth arrest, apoptosis, DNA repair, and/or the eventual development of a senescent phenotype [13,15]. The primary effector of p53 in cellular senescence is believed to be the cdk2/4 inhibitor p21, which by downregulation of E2F prevents the expression of the genes required for S-phase initiation and DNA replication [25]. However, there is increasing evidence with cell differentiation agents, as we have shown with retinoids, to suggest that the p16-pRb (Retinoblastoma protein) pathway is also involved in cellular senescence [17]. Overexpression of p16 apparently inhibits cdk4/6 activity and pRb phosphorylation, and thus suppresses cell cycle progression [14,16]. Our data support the potential role of p16 in mediating the antitumor effects of DHEA and DHEA 8354. In contrast, p53 expression was not affected, as determined by western blotting and ICH (data not shown). Because the animals in this study were treated with DHEA and DHEA 8354 for 6 weeks and then tumors were examined, we cannot exclude the possibility that at early time points the gene encoding p53, or other genes, might also contribute to the development of cellular senescence in mammary tumors.

The exact mechanism by which DHEA exerts its chemopreventive and antitumor effects on mammary carcinogenesis is not known. In animals treated with DHEA an increase in estradiol circulation level has been observed, suggesting more general mechanisms of tumor growth inhibition. This has been also supported by the data from C3(1)/SV40 Tag-transgenic mice, which spontaneously develop ER-negative mammary tumors and were also suppressed by DHEA [9]. DHEA has similarly been found to suppress prostate carcinogenesis in rats [27], despite the fact that it may increase the levels of testosterone and dihydrotestosterone [28]. DHEA is also known to activate peroxisome proliferator-activated receptor (PPAR)-α and PPAR-γ, which may induce differentiation and inhibit carcinogenesis [7].

Our data indicate that DHEA and DHEA 8354 at doses of 1,000 mg/kg diet had the following effects: they decreased the multiplicity and the weight (burden) of mammary tumors; they induced cellular senescence; they inhibited cell proliferation; they arrested the cells in G1/G0 phases of the cell cycle; they increased apoptotic cell death; and they induced the expression of p16 and p21 but not that of p53.

On the basis of our results we propose a model of inhibition of mammary carcinogenesis by DHEA that involves the suppression of cell proliferation, the induction of senescence in tumor cells and the induction of apoptosis (Fig. 5); these cellular events are apparently associated with the upregulation of p16 and p21 but not that of p53, implying a p53-independent mechanism.

## Conclusion

In this study we provide evidence that DHEA and DHEA 8354 suppress the progression phase of mammary carcinogenesis by inducing cellular senescence, inhibiting cell proliferation and increasing apoptotic cell death. We also found that p16^INK4A ^and p21, but not p53, are upregulated in mammary tumors of animals treated with high doses of DHEA, suggesting a p53-independent mechanism of tumor suppression.

## Abbreviations

BrdU = bromodeoxyuridine; DHEA = dehydroepiandrosterone; ER = estrogen receptor; ICH = immunocytochemical assay; MNU = *N*-nitroso-*N*-methylurea; SA-β-Gal = senescence-associated β-galactosidase.

## Competing interests

The authors declare that they have no competing interests.

## Authors' contributions

AS performed the immunohistological and pathological studies. AG maintained the animals and performed surgical procedures at appropriate times. VP performed p53, p21, p16, and cell-cycle-related studies. VS and RL helped in designing the study, in critical analysis of results and in comments on the manuscript. KC developed the concept of the study, implemented the SA-β-Gall and other assays for identification of senescent cells, supervised the experimental work and the interpretation of results and contributed to writing the manuscript. All authors read and approved the final manuscript.

## Figures and Tables

**Table 1:** Effects of dehydroepiandrosterone (DHEA) and DHEA 8354 on mammary tumor development

Treatment	Dose (mg/kg diet)	No. of animals	Weight of animals (g)	Tumor incidence (%)	Tumor multiplicity	Tumor (weight) burden (g)
Control	0	20	234	93.3	4.0	5.7
DHEA	125	29	231	96.7	3.2^a^	3.7^b^
	1,000	30	218	76.7	2.3^a^	2.3^b^
DHEA 8354	125	30	227	87.6	3.6	4.7
	1,000	29	222	100.0	3.4^a^	2.6^b^

^a^Significant difference for the frequency of tumors, DHEA (*p *< 0.01 and *p *< 0.001) and DHEA 8354 (*p *< 0.05). ^b^Significant difference for tumor weight/g (*p *< 0.01 and *p *< 0.01, DHEA and DHEA 8354, respectively) over the corresponding control (MNU-treated) groups.

**Table 2:** Effects of DHEA and DHEA 8354 on SA-β-Gal cells, BrdU-labeled cells and apoptosis

Treatment	Dose (mg/kg diet)	No. of tumors^a^	SA-β-Gal cells (%)	BrdU-labelled cells (%)	Apoptosis (%)
Control	0	10	0.4 ± 0.2	15.6 ± 4.8	0.7 ± 0.4
DHEA	125	9	1.9 ± 1.8^b^	9.9 ± 4.2^c^	1.6 ± 0.4^d^
	1,000	15	3.3 ± 2.0^b^	2.2 ± 1.2^c^	3.2 ± 2.0^d^
DHEA 8354	125	8	0.7 ± 0.4	13.5 ± 4.4	1.1 ± 0.4
	1,000	10	2.8 ± 1.0^b^	5.3 ± 1.8^c^	1.5 ± 0.5^d^

^a^One tumor per animal was examined once; ^b,c,d^the differences in values between treated and control tumors are significant (*p *< 0.05; Student's *t *test). BrdU, bromodeoxyuridine; DHEA, dehydroepiandrosterone; SA-β-Gal, senescence-associated β-galactosidase (mean ± SD%) values.

**Table 3:** Effects of DHEA and DHEA 8354 on the cell cycle phases (flow cytometry data)

Treatment	Dose (mg/kg diet)	No. of tumors	Phase of the cell cycle		
			
			G1/G0 (%)	S (%)	G2M (%)
MNU	-	10	92.3 ± 0.8	5.4 ± 2.2	2.4 ± 1.1
DHEA	1,000	11	95.9 ± 1.8^a^	1.8 ± 0.8^b^	2.1 ± 1.5
DHEA 8354	1,000	14	94.8 ± 1.7^a^	2.1 ± 1.0^b^	2.3 ± 1.1

^a,b^Significant (*p *< 0.001) differences from control tumors. DHEA, dehydroepiandrosterone; MNU, *N*-nitroso-*N*-methylurea (mean ± SD) values.

**Table 4:** Effects of dehydroepiandrosterone on p16 and p21 expression in mammary tumors (immunohistochemical assay)

Group	No. of animals	p16		P21	
		Expression^a^	*p*	Expression^a^	*p*
Control	10	3.5 ± 2.2		5.6 ± 1.3	
Dehydroepiandrosterone^b^	8	6.3 ± 2.2	0.01	6.1 ± 1.2	n.s.

^a^p16 and p21 values were generated semiquantitatively as described in the Materials and methods section; ^b^dehydroepiandrosterone was given at 1,000 mg/kg.
